# Healthcare professionals’ perceptions about interprofessional teamwork: a national survey within Swedish child healthcare services

**DOI:** 10.1186/s12913-021-06139-3

**Published:** 2021-03-22

**Authors:** Ulrika Svea Nygren, Ylva Tindberg, Leif Eriksson, Ulf Larsson, Håkan Sandberg, Lena Nordgren

**Affiliations:** 1 Department of Public Health and Caring Sciences, Uppsala University, BMC, Box 564, 751 22 Uppsala, Sweden; 2 Center for Clinical Research Sörmland/Uppsala University, Box 529, 631 07 Eskilstuna, Sweden; 3 Department of Women’s and Children’s Health, Uppsala University, Akademiska sjukhuset, 751 85 Uppsala, Sweden; 4 School of Health, Care and Social Welfare, Mälardalen University, Box 883, 721 23 Västerås, Sweden

**Keywords:** Child healthcare services, Interprofessional teamwork, Team-based visit, Parental groups, Family center, Cross-sectional survey

## Abstract

**Background:**

Globally, interprofessional teamwork is described as a key method to promote health and prevent illness in children, namely, to achieve the goals of Child Healthcare Services (CHS). However, how teamwork should be designed within CHS to achieve the goals is unclear. This study aimed to investigate healthcare professionals’ perceptions about 1) taking part in interprofessional teamwork, 2) team characteristics, and 3) whether the perceptions were related to professional affiliation or workplace.

**Methods:**

A national cross-sectional survey was conducted using a web-based study-specific questionnaire sent to all accessible nurses, physicians, and psychologists in Swedish CHS (*n* = 3552). The response rate was 31.5%. To identify possible associations, logistic regressions were conducted.

**Results:**

Almost all respondents, 1096/1119 (97.9%), reported taking part in some type of interprofessional teamwork within the Swedish CHS. Among those, the most common was team-based visits (82.2%). It was perceived that performing team-based visits resulted in fulfilled goals, expertise exceeding individual team members’ competences, provision of high-quality care, and meeting children’s and families’ needs, to a greater extent, than if not performing team-based visits. Correspondingly, working as a team in parental groups was perceived as resulting in fulfilled goals, meeting the needs of children and their families, and continuity within the team to a greater extent than if not working together in a team. Professional affiliation was associated with different perceptions and types of teamwork. Family Centers were positively associated with all types of teamwork as well as continuity within the team.

**Conclusions:**

Healthcare professionals’ perceptions about team characteristics were associated with professional affiliation, workplace, and type of teamwork (defined as team activities) within the CHS. Professionals within Swedish CHS, taking part in team-based visits and in interprofessional teamwork in parental groups, perceived that the team fulfilled its goals and met the needs of children and families to a greater extent than professionals not taking part in these types of teamwork. Professionals at Family Centers were more likely to work in teams in different ways.

Knowledge about interprofessional teamwork for individuals and groups in Swedish CHS might also be valuable in other healthcare settings, dealing with complex needs.

## Background

In 2014, new instructions for Swedish Child Healthcare Services (CHS) were published in which extended interprofessional teamwork was prescribed as a key method [1]. At present, however, knowledge is lacking about how interprofessional teamwork should be designed in order to achieve the goals of the CHS, that is, to meet the needs of children and their families. In order to develop more knowledge, we asked professionals within the Swedish CHS about their perceptions of teamwork. The main question was: how do healthcare professionals within the Swedish CHS perceive different types of teamwork, i.e., team activities, including prerequisites and effects of teamwork? We also wanted to understand whether their perceptions were related to their professional affiliation or to their workplace.

All children have the right to the best possible health and to good quality healthcare [1, 2]. The aim of CHS is to promote children’s health and development and to prevent illness by reducing the influence of risk factors as well as strengthening protective factors in and around the child [1, 3, 4]. Globally, CHS is an important health arena [5]; moreover, there is evidence showing that National Child Health Programs (NCHP) are beneficial for health and longevity [6]. Despite major progress during the last few decades regarding children’s health and access to CHS, there is still a lot of work to be done concerning the implementation of evidence-based interventions within CHS [5]. International comparisons between NCHPs show variations concerning intensity, quality, content, and number of child health visits. In addition, there are differences regarding professions and organizational contexts, affecting the coverage and outcome of CHS [3–5, 7, 8]. According to Wood et al. [3], the variations in CHS are likely to be caused by differences in local priorities or by the lack of evidence regarding different activities within NCHPs [3, 4]. Furthermore, Alexander et al. [7] state that it is problematic to determine from previous research reports exactly “who” has delivered and “what” they have delivered. Globally, to achieve the goals of CHS, there is a need for research regarding methods, outcomes, and involved healthcare professionals [3, 4, 7].

The Swedish NCHP is constituted by instructions for CHS published by the National Board of Health and Welfare, together with a web-based national guide [1, 9]. According to the NCHP, interprofessional teamwork is recommended in the shape of consultations, i.e., when different professionals seek advice from each other regarding a child or his or her family, without the child or the family being present [1, 9]. The NCHP also recommend team-based visits, i.e., when different professionals see the child and his or her family in joint physical meetings. Team-based visits involve different combinations of professionals, depending on the purpose of the visit [1, 9]. Teamwork can imply different healthcare professionals being represented in a parental group, or different professionals meeting for the exchange of experiences and knowledge in so-called team meetings, or other types of teamwork such as joint home visits and collaborative education [9, 10].

The World Health Organization [11] describes how healthcare professionals from different backgrounds should work together with families to deliver the highest quality of care and to achieve the health goals. There is evidence that teamwork is important for an effective healthcare [12–14] to meet the patients’ needs [12–16], and to promote equal health and development for children [17, 18]. The concepts ‘interprofessional teamwork’ and ‘interprofessional collaboration’ are often used interchangeably, which has been problematized in previous research [13–15, 19]. The different conceptual interpretations for teamwork [13–15, 19] also concern studies regarding CHS [20–23]. Reeves et al. [12, 19] distinguish between interprofessional teamwork and other forms of interprofessional practice [12, 19]. Interprofessional teamwork does not occur simply by putting different professionals together [12, 13, 19, 24]. According to the literature [12–16, 19, 24–29], certain prerequisites and effects are needed for a working group to be characterized as a team.

Prerequisites for a team:

- Shared goals [13–15, 19, 24, 25]
- Patient focus (child and family focused) [13, 15, 28]
- Equal relationships within the team [16, 27, 29]
- Effective organization [12, 15, 27, 28]
- Continuity [12, 15, 16]

Team effects:

- Fulfilled goals [12, 15, 19, 25, 27]
- Expertise that exceeds the competence of individual team members [24, 27]
- High-quality care [13, 15, 25, 27]
- Meet the needs of the patient (child and family) [13, 15, 16, 27]

Clements et al. [13] and Drinka et al. [15] described teamwork as a process that has the quality of being a support within itself. Teamwork and team characteristics are interdependent [13, 15]. From a system theoretical perspective [28], CHS, where children and families meet healthcare professionals, can be defined as a clinical microsystem. A clinical microsystem is the smallest unit in the healthcare system, surrounded by meso- and macrosystems [28]. Contextual factors within the micro-, meso-, and macrosystems that influence teamwork include those on the individual, organizational, and societal levels [15, 28, 30, 31]. Individual factors include profession, and the individuals’ experiences and perceptions [32, 33]. Organizational factors include physical settings, workplaces, local instructions, and resources [15, 28, 30]. Social factors include politics, national instructions, and resources [15].

To the best of our knowledge, there is no published research about CHS that defines teamwork in line with the Swedish NCHP. Furthermore, how teamwork within CHS should be designed to achieve the goals of the CHS is unknown. To develop knowledge about how to organize optimal teamwork, an important step is to understand how healthcare professionals perceive teamwork in CHS. Earlier studies have shown that team members’ expressions of collaborative quality give clear indications of what successful teamwork entails. The team members’ perception of collaboration is a way of getting a hint of team efficiency [15, 34]. This study is part of a larger research project that aims to produce evidence-based knowledge, grounded on nurses,’ physicians,’ and psychologists’ perceptions about teams and interprofessional collaboration within the Swedish CHS setting. The present study aimed to investigate healthcare professionals’ perceptions about 1) taking part in interprofessional teamwork, 2) team characteristics, and 3) whether the perceptions were related to professional affiliation or workplace.

## Methods

### Design

A national cross-sectional survey was conducted using a web-based study-specific questionnaire targeting all accessible nurses, physicians, and psychologists in the Swedish CHS.

### Setting

In Sweden, all children have the right to free CHS through regular visits at Child Healthcare Centers (CHC) from birth until school start at age six. The provision and financing of CHS are public matters, overseen by both private and public providers [35]. The CHS is organized by 21 regions, which are all autonomous and decide locally on priorities [35]. The regions are, in turn, merged into six Healthcare Regions that cooperate in specialist healthcare or disciplines such as CHS [35]. In each region, a Main Child Healthcare Unit (MCHU) is responsible for monitoring children’s health and support of professionals and managers at the local CHS. In addition, the managers at the local CHS have personnel, economical, and organizational responsibilities [1, 35, 36]. The CHS are provided in Healthcare Centers, in CHC, or in Family Centers or elsewhere. A Family Center is a co-location for CHS, maternal and parental health care, social services, and open preschool [1, 37]. The CHS are led by public health nurses or pediatric nurses, who collaborate closely with general practitioners, pediatricians, and psychologists [35, 37]. Both nurses and physicians are usually employed by a primary Healthcare Center. However, psychologists are organized differently as they can work at Psychologist Clinic, MCHUs, CHCs, Family Centers, or other places like hospital clinics.

### Data collection

A questionnaire was distributed electronically to all nurses, physicians, and psychologists engaged in Swedish CHS between October 2017 and February 2018 (*n* = 3552). E-mail addresses to the respondents were obtained by healthcare developers at the regional MCHU and from the national Psychologist Association. In some cases, additional approval was required from the county council’s management or Research and Development Units to obtain e-mail addresses. E-mail addresses were forwarded to a digital survey tool, Artologic, which distributed the questionnaire and an accompanying letter. Informed consent was obtained as the questionnaire was answered. After 2 weeks, a first reminder was sent out to non-respondents and after an additional 2 weeks, another reminder was sent out [38]. Anonymity was ensured by sending the answers electronically directly to the survey tool, where the answers were encoded before they were saved in the dataset. The code key was kept separate from the dataset.

### The questionnaire

The specific instrument to be used is quite dependent on the context wherein it is needed [39]. Based on a comparison of available questionnaires [40], we chose one developed by Thylefors et al. [14, 27] regarding the questions about team characteristics. However, for the questions about teamwork, comprising team activities, a context specific questionnaire had to be developed. Based on the interplay between theory and field research, the study-specific questionnaire was developed by a research group with competences, including experiences of clinical CHS, regional and national CHS development, research on teams, and knowledge of questionnaire design. Before distribution, the questionnaire was tested for content validity by the healthcare developers (*n* = 8), medical directors (*n* = 2), and the psychologists at the regional MCHU (*n* = 10). Minor corrections were made to fit the entire country as well as nurses, physicians, and psychologists. Before its final release, the questionnaire was tested with a group of 15 participants consisting of healthcare developers and doctoral students [38]. For *content validity,* teamwork according to the NCHP was described in detail in the survey, with further clarifications following the pilot [38]. Information on teamwork was provided together with the final questionnaire containing 13 questions with additional follow-up questions.

The questionnaire collected predominantly quantitative data through questions with fixed response alternatives. To avoid missing any answers, most questions were compulsory and free space was provided for comments. Questions about individual factors concerned sex, profession (nurse, physician, and psychologist), and years in the CHS. Organizational factors included workplace and number of current workplaces (Table 1). Before analysis, the multiple-choice question about the respondents’ workplace was transformed into three categories: Family Center, CHCs, or Other (including Psychologist Clinic, MCHU, Healthcare Center, Specialist CHS). The number of workplaces was categorized as 1, 2, or > 2.

**Table 1:** Characteristics of study population

Individual factors	Number	(%)
**Sex**		
Female	1029	(92.6)
Male	83	(7.4)
**Profession**		
Nurse	741	(66.2)
Physician	234	(20.9)
Psychologist	144	(12.9)
**Years in Child Healthcare Services**		
< 6	407	(36.4)
6–20	521	(46.6)
> 20	191	(17.0)
**Organizational factors**		
**Number of current workplaces**		
1	879	(78.5)
2	86	(7.7)
Other	154	(13.8)
**Workplace**		
Child Healthcare Center	669	(59.8)
Family Center	264	(23.6)
Other	186	(16.6)
**All**	**1119**	**(100.0)**

Information on the respondent’s interprofessional teamwork was obtained from a multiple-choice question worded as follows: “Do you work in any of the following ways in a team at the CHS?” The response options were: Team-based visits (joint physical meetings between different professionals, the child, and his or her family), Consultation regarding an individual child and/or his or her family, Teamwork with parental groups, Meetings for exchange of experiences and knowledge (Consultation and guidance), Other types of interprofessional teamwork, and ‘No, I work on my own’.

For respondents who answered that they worked in a team, there were follow-up questions related to perceptions about the team. They were asked to rate on a 5-point Likert scale ranging from “To a very high degree” to “To a very low degree” the extent to which they perceived that the team worked toward shared goals, held equal relationships within the team, were efficiently organized, stood for continuity, fulfilled its goals, and had expertise that exceeded the competence of the individual team members. Expertise exceeding the sum of individual team members’ competence was used as a proxy for synergy effects. The respondents were also asked to rate the extent to which they perceived that the team provided high-quality efforts and met the needs of children and their families (Textbox 1). All questions regarding team characteristics, except those about perceived continuity and equality within the team, were based on Thylefors et al. [14, 27]. The reliability has been estimated by Cronbach’s alpha (α = 0.91) [14, 27]. Response options for rating the “team characteristics” were dichotomized as: 1 ([yes] = “to a very high degree” and “to a high degree” or 2 ([no]= “to neither a high nor low degree,” “to a low degree,” and “to a very low degree”).

### Statistical analysis

Binary (Yes/No) logistic regressions were performed for three separate models. In a first model, associations between individual and organizational factors were tested against different types of teamwork. In a second model, associations between different types of teamwork and perceptions about prerequisites and effects of the team were tested. In a third model, professional affiliation and workplace were tested against perceptions about team characteristics. In all models, adjustments were made for sex, profession, years in CHS, and workplace when appropriate. The results are reported as adjusted odds ratios with 95% confidence intervals and *p-*values. The significance level was set at *p* < .05. All analyses were performed with IBM SPSS Statistics 22.0.

### Ethical approval

Informed consent was obtained electronically from all participants answering the questionnaire. The study and the consent procedure were reviewed and approved by the Regional Ethics Review Board in Uppsala (Dnr 2017/356).

## Results

### Characteristics of respondents

In total, 1119 (31.5%) professionals responded to the questionnaire, of whom a majority were women (92.6%) (Table 1). The largest group of respondents were nurses (66.2%), followed by physicians (20.9%), and psychologists (12.9%). Six out of ten participants had worked for more than six years within CHS (63.6%), and a majority (78.5%) reported only one current workplace. The most reported localizations for providing CHS were in CHCs (59.8%) and in Family Centers (23.6%) (Table 1).

### Types of teamwork

Almost all respondents (1096/1119; 97.9%) participated in teamwork. Team-based visits were the most common type (82.2%), followed by consultations (77.9%), team meetings (65.2%), parental groups (42.3%), and other non-specified types of teamwork (17.0%).

As shown in Table 2, professional affiliation was significantly associated with the different types of teamwork. Respondents who worked at Family Centers reported participation in all types of teamwork, except other non-specified types of teamwork, to a significantly greater extent than respondents who worked at the CHC (Table 2).

**Table 2:** Associations between different types of teamwork within Child Healthcare Services and individual and organizational factors, respectively

	All respondents	Team-based visits				Consultations				Team meetings				Parental groups				Other			
Individual factors^c^	n	n	(%)	AOR^a^	*p*-value	n	(%)	AOR^a^	*p*-value	n	(%)	AOR^a^	*p*-value	n	(%)	AOR^a^	*p*-value	n	(%)	AOR^a^	*p*-value
**Profession**																					
Nurse	741	663	(89.5)	Ref		606	(81.8)	Ref		414	(55.9)	Ref		560	(75.6)	Ref		137	(18.5)	Ref	
Physician	234	203	(86.8)	0.8 (0.5-1.2)	.22	137	(58.5)	0.3 (0.2-0.4)	<.001	15	(6.4)	0.1 (0.0-0.1)	<.001	43	(18.4)	0.1 (0.1-0.1)	<.001	27	(11.5)	0.6 (0.3-0.9)	.009
Psychologist^b^	144	54	(37.5)^b^	0.1 (0.1-0.1)	<.001	129	(89.6)	2.1 (1.2-3.7)	.02	44	(30.6)	0.4 (0.2-0.5)	<.001	127	(88.2)	2.4 (1.4-4.0)	.001	26	(18.1)	1.0 (0.6-1.6)	.98
**Organizational factors**^d^																					
**Workplace**																					
Child Healthcare Center	669	575	(85.9)	Ref		497	(74.3)	Ref		261	(39.0)	Ref		407	(60.8)	Ref		108	(16.1)	Ref	
Family Center	264	240	(90.9)	1.9 (1.2-3.2)	.01	222	(84.1)	1.7 (1.2-2.6)	.005	160	(60.6)	2.5 (1.8-3.5)	<.001	191	(72.3)	1.6 (1.1-2.3)	.01	48	(18.2)	1.1 (0.8-1.6)	.56
Other	186	105	(56.5)	0.8 (0.5-1.4)	.48	153	(82.3)	1.1 (0.7-1.9)	.62	52	(28.0)	0.8 (0.5-1.3)	.42	132	(71.0)	0.9 (0.5-1.5)	.75	34	(18.3)	1.3 (0.7-2.2)	.36

^a^Adjusted odds ratio (AOR)

^b^Psychologists do not participate in universal team-based visits according to the National Child Healthcare Program, just in targeted team-based visits on specific indications

^c^Adjusted for sex and years in Child Healthcare Services

^d^Adjusted for sex, profession and number of current workplaces

### Perceptions about the team

When looking at perceptions of prerequisites for teamwork, the vast majority reporting any type of interprofessional teamwork perceived that the team worked toward shared goals (93.4%), had an equal relationship within the team (80.0%), stood for continuity (80.0%), and was efficiently organized (68.4%). Furthermore, most of the respondents perceived effects of teamwork such as: fulfilled goals (82.9%), expertise within the team exceeded the sum of individual team members’ competences (78.7%), high-quality efforts (90.7%), and met the needs of children and their families (88.7%).

Respondents reporting team-based visits rated all teamwork effects to a significantly greater extent than respondents who did not participate in team-based visits (Table 3). Regarding prerequisites for teamwork, respondents who participated in team-based visits responded that the team worked toward shared goals, had equal relationships within the team, and was efficiently organized, to a significantly greater extent than respondents who did not participate in team-based visits (Table 3). Respondents who worked in teams with parental groups reported that the team met the needs of children and their families, fulfilled its goals, and stood for continuity to a significantly greater extent than respondents who did not work in teams with parental groups (Table 3).

**Table 3:** Associations between perceptions of team characteristics (prerequisites for team and team effects) and type of teamwork among professionals within Child Healthcare Services

**Prerequisites for team**												
	**Shared goals**			**Equality**			**Efficiency organization**			**Continuity**		
**Type of teamwork**^b^	**(%)**	**AOR**^a^	***p*-value**	**(%)**	**AOR**^a^	***p*-value**	**(%)**	**AOR**^a^	***p*-value**	**(%)**	**AOR**^a^	***p*-value**
**Team-based visits**												
No	(86.4)	Ref		(60.8)	Ref		(43.8)	Ref		(71.6)	Ref	
Yes	(94.8)	2.0 (1.1–3.7)	.03	(83.7)	2.0 (1.3–3.1)	.001	(73.1)	2.6 (1.8–3.8)	<.001	(81.6)	1.4 (0.9–2.2)	.11
**Consultations**												
No	(93.8)	Ref		(81.7)	Ref		(76.8)	Ref		(80.8)	Ref	
Yes	(93.3)	1.1 (0.6–2.1)	.80	(79.6)	1.2 (0.8–1.8)	.42	(66.2)	0.7 (0.5–1.0)	.08	(79.8)	1.0 (0.7–1.6)	.84
**Parental groups**												
No	(92.8)	Ref		(80.9)	Ref		(66.9)	Ref		(78.3)	Ref	
Yes	(94.3)	1.5 (0.9–2.5)	.17	(78.8)	0.8 (0.6–1.2)	.67	(70.3)	1.3 (1.0–1.8)	.08	(82.2)	1.5 (1.1–2.1)	.01
**Team meetings**												
No	(94.0)	Ref		(83.6)	Ref		(75.4)	Ref		(82.2)	Ref	
Yes	(93.1)	1.4 (0.8–2.6)	.30	(78.2)	1.2 (0.8–1.8)	.28	(64.9)	0.8 (0.6–1.2)	.24	(78.9)	1.0 (0.7–1.5)	.83
**Team effects**												
	**Fulfilled goals**			**Expertise**			**High quality**			**Meet needs of the child**		
**Type of teamwork**^b^	**(%)**	**AOR**^a^	***p*-value**	**(%)**	**AOR**^a^	***p*-value**	**(%)**	**AOR**^a^	***p*-value**	**(%)**	**AOR**^a^	***p*-value**
**Team-based visits**												
No	(68.2)	Ref	<.001	(68.2)	Ref	.001	(83.0)	Ref	.008	(79.0)	Ref	.003
Yes	(85.7)	2.3 (1.5–3.6)		(80.7)	2.0 (1.3–3.1)		(92.2)	2.1 (1.2–3.6)		(90.5)	2.1 (1.3–3.5)	
**Consultations**												
No	(85.3)	Ref	.69	(78.6)	Ref	.42	(92.4)	Ref	.65	(88.4)	Ref	.58
Yes	(82.3)	0.9 (0.6–1.4)		(78.8)	1.2 (0.8–1.8)		(90.2)	0.9 (0.5–1.6)		(88.7)	1.2 (0.7–1.9)	
**Parental groups**												
No	(81.2)	Ref	.03	(79.3)	Ref	.74	(90.4)	Ref	.26	(87.0)	Ref	.02
Yes	(85.2)	1.5 (1.0–2.1)		(78.0)	0.9 (0.7–1.3)		(91.1)	1.3 (0.8–2.0)		(90.9)	1.7 (1.1–2.6)	
**Team meetings**												
No	(85.2)	Ref	.88	(80.9)	Ref	.95	(91.8)	Ref	.42	(89.6)	Ref	.75
Yes	(81.8)	1.0 (0.7–1.6)		(77.6)	1.0 (0.7–1.5)		(90.1)	1.2 (0.7–2.1)		(88.2)	1.1 (0.7–1.8)	

^a^Adjusted Odds Ratio (AOR)

^b^Adjusted for sex, profession and all other types of teamwork

Professional affiliation was associated with perceptions of the team as shown in Table 4. Physicians reported fulfilled prerequisites to a significantly greater extent than nurses in the case of shared goals, equality within the team, and efficiently organized teams. Furthermore, physicians rated positive effects of the team to a significantly greater extent than nurses. Psychologists, on the other hand, rated fulfilled prerequisites and positive effects of teamwork to a significantly lower extent compared to nurses.

**Table 4:** Associations between perceptions of team characteristics (prerequisites for team and team effects) and contextual factors (individual and organizational) among professionals within Child Healthcare Services

**Prerequisites for team**												
	**Shared goals**			**Equality**			**Efficient organization**			**Continuity**		
**Individual factors**^b^	**(%)**	**AOR**^a^	***p*-value**	**(%)**	**AOR**^a^	***p*-value**	**(%)**	**AOR**^a^	***p*-value**	**(%)**	**AOR**^a^	***p*-value**
**Profession**												
Nurse	(93.7)	Ref		(81.6)	Ref		(69.7)	Ref		(79.5)	Ref	
Physician	(97.3)	3.6 (1.3–10.3)	.02	(90.6)	2.7 (1.5–5.0)	.001	(79.0)	1.6 (1.1–2.4)	.02	(87.9)	1.5 (1.0–2.5)	.08
Psychologist	(85.8)	0.4 (0.2–0.8)	.004	(54.6)	0.3 (0.2–0.5)	<.001	(44.7)	0.4 (0.3–0.5)	<.001	(70.2)	0.6 (0.4–0.9)	.02
**Organizational factors**^c^												
**Workplace**												
Child Healthcare Center	(94.8)	Ref		(83.3)	Ref		(70.5)	Ref		(80.2)	Ref	
Family Center	(93.5)	0.8 (0.5–1.5)	.54	(81.7)	1.0 (0.7–1.4)	.87	(71.4)	1.1 (0.8–1.5)	.58	(85.5)	1.5 (1.0–2.3)	.04
Other	(88.5)	0.7 (0.3–1.6)	.43	(65.9)	0.8 (0.5–1.3)	.34	(56.6)	1.0 (0.6–1.5)	.86	(71.4)	0.7 (0.4–1.2)	.20
**Team effects**												
	**Fulfilled goals**			**Expertise**			**High quality**			**Meet needs of child and family**		
**Individual factors**^b^	**(%)**	**AOR**^a^	***p*-value**	**(%)**	**AOR**^a^	***p*-value**	**(%)**	**AOR**^a^	***p*-value**	**(%)**	**AOR**^a^	***p*-value**
**Profession**												
Nurse	(83.2)	Ref		(77.7)	Ref		(90.3)	Ref		(88.6)	Ref	
Physician	(89.7)	1.7 (1.0–2.9)	.05	(85.3)	1.8 (1.1–3.0)	.01	(95.5)	3.9 (1.6–9.4)	.002	(93.3)	2.0 (1.0–3.9)	.05
Psychologist	(70.9)	0.5 (0.3–0.7)	.001	(73.8)	0.8 (0.5–1.3)	.37	(85.1)	0.7 (0.4–1.2)	.17	(81.6)	0.6 (0.4–1.0)	.07
**Organizational factors**^c^												
**Workplace**												
Child Healthcare Center	(84.2)	Ref		(78.3)	Ref		(91.6)	Ref		(89.2)	Ref	
Family Center	(85.1)	1.1 (0.7–1.7)	.59	(81.3)	1.2 (0.8–1.8)	.84	(92.0)	1.1 (0.6–1.9)	.74	(92.0)	1.4 (0.8–2.3)	.20
Other	(75.3)	0.8 (0.5–1.4)	.44	(76.4)	1.0 (0.6–1.6)	.58	(85.7)	0.6 (0.3–1.2)	.16	(81.9)	0.6 (0.3–1.1)	.13

^a^Adjusted Odds Ratio (AOR)

^b^Adjusted for sex and years within Child Healthcare Services

^c^Adjusted for sex, profession and number of current workplaces

Respondents who worked at Family Centers perceived that the team stood for continuity to a significantly greater extent than respondents who worked at the CHC (Table 4).

## Discussion

This cross-sectional study contributes with knowledge about healthcare professionals’ perceptions about interprofessional teamwork within the Swedish CHS. The results show that a vast majority of the respondents perceived that they worked in teams.

Our main finding is that professionals in CHS, who take part in team-based visits and in interprofessional teamwork in parental groups, perceive, to a greater extent, that the team fulfills its goals and meets the needs of children and families compared to professionals that do not take part in these types of teamwork. In the Swedish NCHP, team-based visits are defined as physical meetings between different healthcare professionals, the child, and the family [1, 9]. In previous studies regarding teamwork in CHS, teamwork has been defined as “shared care,” implying that nurses and physicians provide separate activities [3, 20–23]. Nelson et al. [28] describe a clinical microsystem as *living units,* having the person with his or her health needs in the center with professionals coming together to meet the needs of the individual, in this case children and their families. Both team-based visits and teamwork with parental groups are physical meetings, where different professionals meet the child and his or her family in the same place and at the same time, with the aim to promote goals and to satisfy the needs of the child and his or her family. The interprofessional teamwork, where medical, psychological as well as social circumstances are taken into account, thus, enabling a holistic view of the child in his or her family [1, 9, 11], seems to be promoted through team-based visits and interprofessional teamwork in parental groups. Consequently, the present study contributes with knowledge about teamwork in the forms of team-based visits and parental groups as possible methods for developing efficient CHS. Our results also highlight the complex interactions between prerequisites for teamwork and different types of teamwork. Shared goals, equality within the team, and an effective organization were found to be prerequisites associated with team-based visits, while continuity in the team was associated with teamwork in parental groups.

### Individual factors

Our study shows differences between professionals’ perceptions about the team and types of teamwork. In general, physicians perceived that the team had prerequisites needed for teamwork as well as positive teamwork effects to a greater extent than nurses. In previous studies, physicians in primary care have been found to be more reluctant to engage in teamwork than nurses [41, 42]. However, Schadewaldt et al. [43] show that exposure to teamwork helps professionals to see the meaning of collaboration. According to the Swedish NCHP, nurses and physicians are prescribed to participate in universal team-based visits at four specific ages [1, 9], which might have contributed to the present finding. Tell et al. [44] describe that for successful implementation of guidelines, such as the new Swedish NCHP, the content and methods within it must match professional needs and be perceived as relevant [44]. Thus, the present results might reflect a change in physicians’ attitudes toward teamwork within CHS, possibly being related to the introduced team-based visits, and their new knowledge and experiences related to this.

Furthermore, the psychologists did not participate in team-based visits to the same extent as nurses and physicians, which can possibly have influenced their perceptions about the team characteristics. The role and responsibilities of the psychologists in the CHS team might not be sufficiently clear in the NCHP and thus does not match the needs or are not viewed as being relevant from the psychologists’ perspective according to the reasoning by Tell et al. [44].

### Organizational factors

Our results show the associations between the organizational context and perceptions about team and teamwork. Professionals working in Family Centers were more likely to report all types of investigated teamwork as well as a perception of continuity within the team. Our findings correspond with previous studies showing the importance of co-location for enabling teamwork [21, 23, 45, 46]. Family Centers are co-locations that engage different healthcare professionals [37], intended to enable healthcare with the child in the center in order to meet the needs of the child and his or her family.

However, only one out of four participants in our study, and as few as one out of six psychologists, worked in Family Centers. Our results show that professionals in the Swedish CHS do not have the same organizational conditions to participate in team-based visits. Limitations to teamwork can be found in the professional’s own views of their roles, but also in management and organizational limitations, such as lack of structures or resources for teamwork [47]. Pullon et al. [46] describe the importance of the physical environment and structural models for collaborative patient-centered care for those with complex healthcare needs. In the Swedish NCHP, targeted team-based visits that engage different combinations of professionals [1, 9], and where CHS psychologists play their role, are not as clearly organized or described as the universal team-based visits involving nurses and physicians. MacNaughton et al. (2013) describe how physical distance to other team members could impact team members’ ability to interact with each other. Proximity to other team members allows for frequent informal meetings [48]. Thus, to support teamwork between healthcare professionals in the Swedish CHS, which enables a holistic view as described by the NCHP [1, 9], there is a need for improved organizational conditions on several levels.

### Shared goals and goal fulfillment

Most of the healthcare professionals working in teams in this study perceived that the team worked toward shared goals. Nearly as many perceived that one effect of teamwork was fulfilled goals. Shared goals and the team’s goal-fulfillment were positively associated with taking part in team-based visits. Compared to nurses, physicians perceived that the team shared goals and fulfilled its goals to a significantly greater extent, while psychologists perceived that the team shared goals and fulfilled its goals to a significantly lower extent. The goal of CHS is to promote children’s health and development according to their needs [1]. Shared goals and responsibility to fulfill the team’s goals relate to the professionals’ specific skills [13]. Furthermore, goal-orientation is essential for effective teamwork [12, 13, 16, 24, 49]. An international comparison shows that in countries with nurse-led provision of CHS, the focus is more on child development and well-being than in countries where physician-led provision of CHS where the focus is more on the children’s physical health [3]. Reeves et al. [19] argue that practitioners who work together need to reflect on their main purpose(s) and how they can respond to the needs of the client [19]. Hallberg [50] describes difficulties because of the shifting focus in the CHS, from physical health to psychosocial health. The difficulties concern the shift from descriptions of concrete and well-defined duties to more abstract and general descriptions of tasks which are, by definition, open to interpretation [50]. Globally, the aim of CHS is to promote children’s health and development, but there are differences concerning methods and means [1, 3, 4, 7]. The aim of CHS is clear, but the team’s shared tasks are more difficult to interpret. Different professionals can have different expectations concerning the children’s health. Hence, team-based visits might be an arena that promotes shared goals within the CHS. Shared goals can also be a reason for team-based visits and further goal fulfillment.

### Equality

Respondents participating in team-based visits perceived that there was equality within the team to a greater extent than respondents who did not work with team-based visits. Equality concerning shared decision-making and responsibilities is a core component of teamwork [12, 27, 29]. The greatest obstacle for the implementation of effective teamwork within healthcare is the hierarchical culture [13]. Team-based visits might be a way to achieve the full potential of all team members, while equality within the team, conversely, might facilitate team-based visits.

### Effective organization

Team-based visits were positively associated with an effectively organized team. It has been shown that organizational support is needed to provide recognition, enable resources, and legitimize the work of the microsystem [13]. Pullon et al. [46] argue for the importance of structural models as well as physical settings to facilitate opportunities for frequent interprofessional communication and shared goals [15, 28, 51]. Universal team-based visits to all children at specific ages imply a structural model for the Swedish CHS. Our results show the importance of concrete and well-defined tasks as well as the need for improved structure for teamwork within CHS. This is in accordance with Hallberg et al. [50], highlighting the importance of both clarity in national guidelines, but also necessary organizational conditions.

### Continuity

In our study, respondents participating in teamwork with parental groups perceived that the team stood for continuity to a greater extent than respondents who did not participate in this type of teamwork. Frequent interactions and effective communication among team members are essential for teamwork; however, both time and structure are required for this [13, 15]. Scheduled team interactions contribute continuity within the team [48]. In universal team-based visits, physicians and nurses meet each other, the child, and his or her families several times during the child’s first years, which contribute to continuity. Furthermore, working at a Family Center was associated with a perception of continuity within the team. Open communication and consultation could be promoted through continuity, and team-based delivery of care in the same locations [12, 46, 52]. Patient centered care and communication between professionals enable continuity [21]. Family Centers are organized around the child and his or her family and seem to enable the continuity within the team.

### Expertise and high quality

In the present study, almost eight out of ten participants perceived that the team had effects of synergy and nine of ten perceived effects of high quality. Respondents who participated in team-based visits perceived that the team had outcomes such as high quality and expertise to a greater extent than respondents who did not participate in team-based visits. High quality and synergy effects are important outcomes of teamwork [13, 24, 25]. A working group might be in action without prerequisites as shared goals or synergy effects, but not as a team [24].

The CHS focus on health promotion in early childhood, which implies a need for complementary skills and competences [53]. The World Health Organization [11] and Reeves et al. [19] describe that professionals who collaborate closely with the child and his or her family can solve unpredictable and complex tasks, deliver high quality of care [12, 19], learn from each other, and develop team competences [15]. Our results indicate that team-based visits can possibly promote this expanded perspective, since “four eyes see more than two eyes”.

### Meeting the needs of children and families

Team-based visits and teamwork with parental groups were both found to be positively associated with meeting the needs of the child and his or her family. This is in line with findings from other studies showing that in complex situations, a high degree of professionalism, including the skill to use each other’s competence adequately, is required [15, 24]. Clements et al. [13] describe that professionals might consider effective teamwork in healthcare as an asset, but for persons in need of healthcare, it is a prerequisite. Patients’ needs are central in teamwork, and the patients are expected to participate in the team [15]. In both team-based visits and teamwork with parental groups, healthcare professionals with complementary perspectives work together in the same place at the same time with the child and his or her family. According to the Swedish instructions for the CHS, teamwork between physicians, nurses, and psychologists enables a holistic view of the child and his or her family, in which medical, psychological as well as social circumstances are recognized and considered. An expanded perspective [11] that facilitates equal health and development in children as well as safe care in cases of complex needs [13, 15, 17, 18] can be achieved by having team-based visits and parental groups. These findings can have implications for other healthcare settings, dealing with complex needs. According to MacNaughton et al. (2013), patients can benefit from collaborative efforts by receiving more holistic care and through better coordination and continuity of health services [48].

### Other types of teamwork

According to previous definitions [12, 13, 19, 24], groups of professionals working together does not always mean working as a team. Therefore, we investigated associations between perceptions about different types of teamwork, prerequisites for teamwork, and effects of teamwork from the perspective of professionals within the Swedish CHS. Consultations and team meetings were not associated with team prerequisites and team effects to the same extent as team-based visits and teamwork in parental groups. According to Reeves et al. [19], consultations and team meetings may be considered as other forms of interprofessional collaboration or as team processes that enable teamwork. Reeves et al. [19] argue for adding this types of other collaboration forms for the development of an effective teamwork.

### Strengths and limitations

Despite widespread use of teamwork in healthcare settings and the new Swedish instructions for the CHS, the knowledge of teamwork within the Swedish CHS is limited. For that reason, we distributed a national web-based survey in order to collect a large amount of data at a low cost and within a short time [38]. Our response rate (31.5%) could be explained by a professional’s high workload, respondent fatigue in answering questionnaires, the surveys length and complexity, or because the survey did not reach the recipients [38, 54]. The response rate is still in line with other surveys [55], where rates between 23.7–89.0% and an average of 37.0% on an organizational level is usual when collecting data via e-mail. The relatively low response rate may reduce the possibility of generalizability. On the other hand, the respondents in our study are representative of the study group [37, 56], which has been confirmed at national meetings with representatives from the MCHU units in Sweden.

A strength of the present study is the development of a context specific questionnaire [39]. *Face validity* was obtained by consulting several experts in the field. Another strength of a questionnaire could be less interviewer bias [38]. Furthermore, our large study size with more than 1100 respondents, has ensured power and allowed complex testing of statistical associations even though qualitative studies are needed to further explore the found associations. Nevertheless, this is a nationwide study, being the first to investigate teamwork in the Swedish CHS. Also, based on our knowledge, there are no previous studies of a similar subject.

## Conclusions

This study is a contribution to the development of evidence-based methods in the Swedish CHS. Our study indicates that teamwork, in the shape of physical meetings such as team-based visits and parental groups held by a team where professionals, the child, and the parents are present at the same time, seems to be important in order to achieve the goals of the interprofessional team and for the perception of meeting the needs of the child and his or her family. Furthermore, Family Centers organized around the children and their families were positively associated with all types of teamwork as well as continuity within the team.

International comparisons between NCHPs show variations in the organizational context. To our knowledge, there are no previous published studies about teamwork within the CHS where teamwork is defined as team-based visits or teamwork in parental groups. The current study contributes knowledge that can be used to effectively organize teams, where both individual and organizational factors are considered.

Qualitative studies are needed to further understand the design of team-based visits and teamwork in parental groups. Such studies might also provide a deeper understanding of the joint physical meeting “face-to-face,” occurring when professionals, the child, and his or her family meet in team-based visits, joint parental groups, and at Family Centers.

Even though this study is context specific to the Swedish CHS, it contributes with knowledge about teamwork for other healthcare settings, dealing with complex needs. The knowledge about the importance of co-location of different professionals for perceived continuity in teamwork is also of interest for teams operating in similar contexts.

## Data Availability

The questionnaire used in this study was developed for a research project that aims to produce evidence-based knowledge about interprofessional teamwork within the CHS. The questions are described in the methods section, and an English version of the questionnaire is available as a supplementary file. The dataset collected and used for the current study is available from the corresponding author on reasonable request.

## Abbreviations

CHC: Child Healthcare Centers
CHS: Child Healthcare Services
MCHU: Main Child Healthcare Unit
NCHP: National Child Health Programs
